# Relationship of Podoplanin and Glutathione S-transferases T1 Expression with Laryngeal Cancer

**Published:** 2012

**Authors:** Deniz Kanliada, Ender Coskunpinar, Kadir Serkan Orhan, Yasemin Musteri Oltulu, Mehmet Celik, Ayse Eren, Ilhan Yaylim, Kemal Deger

**Affiliations:** 1 *Department of ORL and Head&Neck Surgery, Istanbul Faculty of Medicine, Istanbul, Turkey.*; 2 *Department of Molecular Medicine, Institute of Experimental Medicine, Istanbul University, Istanbul, Turkey.*

**Keywords:** Podoplanin, GST-T1, laryngeal carcinoma, biomarker, squamous cell carcinoma

## Abstract

The aim of this study is to determine whether there is a role of podoplanin and glutathione S-transferases T1 (GST-T1) expression in laryngeal squamous cell carcinoma.

The study was completed with 33 patients and gene expression analysis was performed by qRT–PCR. The podoplanin and GST-T1 expression patterns were analyzed to determine their correlation with clinicopathologic parameters of laryngeal cancer.

Of all patients, 20 had supraglottic, and the remaining 13 had glottic laryngeal cancer. Increased expression of podoplanin was found in 14 tumor tissues, but GST-T1 expression was not detected.

Podoplanin expression did not show any prediction for regional metastasis, thyroid cartilage invasion, lymphatic vessel invasion or tumor differentiation for laryngeal cancer, also there were no significant differences in podoplanin expression between glottic and supraglottic regions, but extracapsullar extension is almost statistically significant (p=0,05).

Laryngeal cancer is the only cancer type among all malignancies for which the survival rate decreased in the last decade. Most of the larynx tumors are malign and 95-98% of them are squamous cell carcinomas (SCC) (1). Human podoplanin consists of 162 amino acids, is a 38 k-DA mucin-type transmembrane glycoprotein and the corresponding gene is localized in 1p36.21. Podoplanin is expressed especially in lymphatic endothelial cells, in alveolar type I cells, osteoblasts and peritoneal mesothelial cells, but not in normal vascular endothelial cells (2-5). Thus, the expression levels can be used as a biomarker for lymphangiogenesis (6, 7). Podoplanin also plays an important role in peripheral lung cell proliferation regulation and lymphatic vascular development (8). The podoplanin expression is upregulated in many different human cancers, including squamous cell carcinomas of the oral cavity, lung, cervix, esophagus, skin and also in dysgerminomas of the ovary and granulosa cell tumors, breast tumors, colorectal tumors, melanomas, mesotheliomas, and some tumors of the central nervous system (CNS) (8-16). Increased expression of podoplanin may cause a higher rate of lymph node metastasis (17). In addition, patients with lymph node metastasis and upregulated podoplanin expression had shorter disease-specific survival rate than other patients. According to the diagnosis, 25 % of cases have regional and 8-10% have distant metastasis (17, 18). Podoplanin is frequently expressed in cutaneous head and neck squamous cell carcinoma (HNSCC) and may serve as predictor for regional lymph node metastasis, locoregional recurrence, and clinical outcome (19).

Neck metastasis is one of the most valuable prognostic factors of survival. Laryngeal SCC with the same tumor stages and localizations may have different neck metastasis patterns. This may be due to the molecular structure and the biological behavior of the tumor. Regional metastasis may be related to lymph angiogenesis. Treatment varies according to the tumor stage and localization. Glottic cancers present a better survival rate than supraglottic and subglottic cancers. Five-years survival rates change between 65.7% to 88.6% (20, 21). The most common reason of mortality of laryngeal SCC is the locoregional recurrence (22).

The glutathione S-transferases (GSTs) are an important family of enzymes involved in phase II xenobiotic metabolism that catalyze biosynthesis and metabolism of many substances, including detoxification of exogenous chemical carcinogens, such as aromatic polycyclic hydrocarbons present in the tobacco (23). They comprise four classes of genes (α, μ, π, and θ) and each class includes various genes (24). GST family consists of different classes of enzymes. GST-T1 enzyme in GST T class has its gene located on chromosome 22q11.2 (25, 26). 

It has been shown that individuals carrying the null genotype of GST have significantly reduced activity of this antioxidant enzyme (27, 28) and so have higher levels of intermediates of oxidative metabolism. This genotype is related with many diseases (29, 30). The revealed alterations in expression of GST-T1 enzyme can cause activation of carcinogenic particles or extinction of toxic effects. Therefore, it is thought that GST-T1 enzyme may be an important biomarker for diagnosis of laryngeal cancer. The aim of the study is to determine whether there is a role of podoplanin and GST-T1 in laryngeal SCC.

## Materials and Methods

**Patients**

Thirty six patients diagnosed by histopathological examinations who underwent total or partial laryngectomy operation with or without neck dissection in Istanbul University Faculty of Medicine Department of ORL and Head and Neck Surgery, were included in the study (between November 2010 and November 2011). The patients who received other primary therapies such as radiotherapy or chemotherapy for laryngeal cancer were excluded. Tissue samples were obtained from both healthy adjacent mucosa and the tumor tissue itself during the surgery. They were immediately stored at -80°C until the RNA extraction procedure. The study protocol was approved by both the Ethical Committee of the Istanbul Faculty of Medicine (November 10, 2010 No, 849) and The Scientific Research Projects Coordination Unit of Istanbul University (Project number, 13410).

**Quantitative Real-Time PCR**

Total RNA was extracted from the tissue samples using Roche, High Pure RNA Tissue Kit (Cat. No.12033674001 Roche, GmbH, D-40724 Hilden, Germany) according to the instructions of the manufacturer. RNA samples were quantified using a NanoDrop® ND-1000 spectrophotometer (Thermo Fisher Scientific, Wilmington, Delaware, USA), and their integrity was checked electrophoretically. First strands of the cDNA samples were synthesized by using RT PCR Kit (Cat. No. 11483188001 Roche, GmbH, D-40724 Hilden, Germany)**. **cDNA’s quality was evaluated by podoplanin PCR with the following primers, Forward: 5’ GAA GAG CCA AGG ACA GGT AC 3’, Reverse: 5’ CAA CTT CAT CCA CGT TCACC 3’. Gene expression analysis was performed by quantitative reverse transcription (qRT)–PCR (LightCycler 1.5, Roche, Germany).

The PCR reaction started with a denaturation step at 95°C for 10 minutes (1 cycle), followed by 40 cycles at 95°C for 10 seconds, 60°C for 30 seconds and 72°C for 1 second. Subsequently, a melting curve program was applied with continuous fluorescence measurement. A standard curve for podoplanin templates was generated through serial dilutions of PCR products. Each reaction was performed duplicate. The β-Actin (ACTβ) gene was used as reference for normalization of the gene expression levels. Primer sequences were determined as β-Actin: sense, 5’-GTC TTC CCC TCC ATC GTG-30; antisense, 50- AGG GTG AGG ATG CCT CTC TT-3’. The results were analyzed by the threshold cycle (Ct) numbers as fold-changes and calculated by the 2^∆(∆CT)^method [2geneT-N(Ct)/2 β -ActinT-N(Ct)] (N, matched normal tissue cDNA; T, tumor tissue cDNA). 

**Statistical analysis**

All statistical analyses were performed using the SPSS version 13.0. The relationship between the podoplanin expression statuses and clinicopathologic parameters was analyzed using the Pearson’s chi-square test or Fisher's exact test. All tests were two-sided, and the P values less than 0.05 were considered statistically significant.

## Results

**Fig 1:**
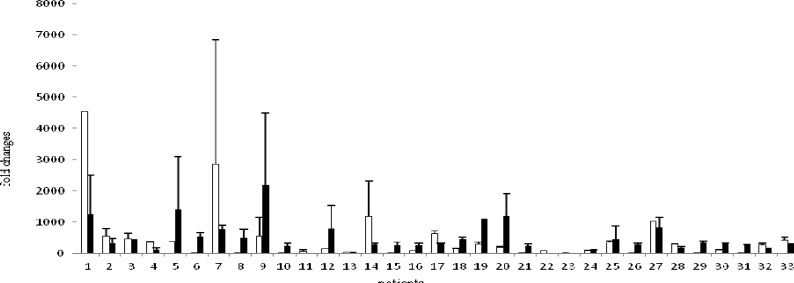
Podoplanin expression in laryngeal cancer patients

Tissue samples were obtained from 42 patients with laryngeal carcinoma, but the tissues of 9 patients were excluded from the study because of technical reasons. Therefore, the study was completed in 33 patients whose mean age±SD was 58.03±11.10 years. All patients were male. Thirty-one patients were smokers, whereas tree patients used alcohol regularly (Table 1). Of all, 20 patients had supraglottic, and the remaining 13 had glottic laryngeal SCC. Podoplanin overexpression was found in 14 patients and on the other hand decreased podoplanin expression was found in 19 patients (Fig.1). The association between the patient characteristics and their podoplanin expressions was shown in (Table 2). GST-T1 expression was not detected.

**Table 1:** Association between podoplanin expression and patient characteristics

Characteristic	Podoplanin Expression Downregulated (n=19) Upregulated (n=14) N % N %				p value
Age					0.622
Mean±SD	54.89±11.18		62.28±9.82		
Median	55		61		
Smoking					1.00*
Yes	18	58.1	13	41.9	
No	1	50	1	50	
Alcohol					0.561*
Yes	1	33.3	2	66.7	
No	18	60	12	40	

*Fisher’s exact test

**Table 2:** Association between Podoplanin expression and clinicopathological data of patients

Characteristic	Podoplanin Expression Downregule (n=19) Upregule (n=14) N % N %				p value
Tumor differentiation					0.948
well	1	50	1	50	
moderately	17	58.6	12	41.1	
poorly	1	50	1	50	
Tumor localization					0.284
Supraglottic tumor	13	65	7	35	
Glottic tumor	6	46.2	7	53.8	
Regional lymph node metastasis					0.241*
Yes	7	77.8	2	22.2	
No	12	50	12	50	
Tumor stage					0.341
T1	0	0	2	100	
T2	8	61.5	5	38.5	
T3	8	66.7	4	33.3	
T4	3	50	3	50	
N stage					0.416*
N0	13	52	12	48	
N1-3	6	75	2	25	
Extra-capsular spread of the lymph nodes					0.05*
Yes	5	100	0	0	
No	14	50	14	50	
Thyroid cartilage invasion					0.212
Yes	4	40	6	60	
No	14	63.6	8	36.4	
Lymphatic vessel invasion					0.486
Yes	8	66.7	4	33.3	
No	11	52.4	10	47.6	

*Fisher’s exact test

## Discussion

Squamous cell carcinoma of the head and neck (HNSCC) is a disease associated with major morbidity and mortality. Recently, it has been reported that podoplanin expression is upregulated in different human cancers, suggesting a role for podoplanin in tumor progression (13-14). However, podoplanin expression alone may not be sufficient to promote tumorigenesis because many of the lesions exhibit the protein expression only in the basal layer cells. Other factors are needed to promote clonal expansion of the abnormal cells. More studies are needed to compare lesions with clonal expansion determined by other markers and podoplanin expression patterns. The data of this study is consistent with recent studies, reporting podoplanin expression in SCCs of different organs (12-13, 17, 31-33). These findings suggest that podoplanin plays a role in the progression of epithelial cancers. The physiological function of podoplanin is still not certain (34). This situation inspires the investigators to find biologic markers to predict the tumoral behavior. Podoplanin expression was investigated in intratumoral and peritumoral tissues of patients with tongue cancer.

Rodrigo et al. found that podoplanin expression was related with regional metastasis that is also supported by our study (34). However, no statistically significant difference about the tumor site was found. Regional lymphatic metastasis observed was two fold higher in patients with low podoplanin expression level than in patients with high podoplanin expression level; but no statistically significant difference was reported. Podoplanin expression levels vary considerably in dysplastic laryngeal epithelium tissue. Therefore, tissue expansion should be observed in multiple regions instead of one region in some cases. Yuan P et al. showed that patients, whose tumors expressed high levels of podoplanin, had a statistically significant higher rate of lymph node metastasis (17)**.** In addition, patients with lymph node metastasis and increased podoplanin expressions had shorter disease-specific survival rate than other patients. Kawaguchi et al. concluded that podoplanin was involved in oral tumorigenesis and may serve as a predictor for lymph node metastasis and poor clinical outcome (35). 

It is well known that the most prognostic factor of laryngeal cancers is regional lymphatic metastasis. Regional metastasis may be related with lymphangiogenesis. For this reason, it was aimed to find if podoplanin expressed on lymphatic vessels but not on the capillary vessels, can be used for the prediction of regional metastasis. Völker et al. reported that the podoplanin expression did not show significant advantages for the prediction of regional nodal metastases in laryngeal and hypopharyngeal SCC (36). Podoplanin expression levels revealed that patients with a significantly poor prognosis in SCC of hypopharynx did not show a significant shorter survival in SCC of laryngeal. Rodrigo et al. showed that the expression of podoplanin in the dysplastic lesions was correlated with the risk of progression to laryngeal cancer (34**).**

The exact molecular function of cancer cell expressed podoplanin is currently studied (37-38). Recent data from studies of various human cancer types suggest a possible association of podoplanin expression with invasion and metastasis of tumors (39-40). Podoplanin expressions significantly decreased as the tumor classification levels increased. Therefore, it was proposed that the podoplanin expression may play a role in the initiation, but not in the progression of laryngeal cancers. Moreover, no relationship was found between the podoplanin expression and the regional nodal metastasis and tumor stage. In this study, extracapsullar extension is almost statistically significant (p=0,05). It is well known that supraglottic and glottic compartments of the laryngeal were developed from different embryologic origin. Glottic region carcinomas are generally well differentiated, and supraglottic region carcinomas are moderate and poor differentiated epidermoid carcinoma. Glottic region carcinoma spreads to anterior commissure with anterior extension, and herefrom spreads to ventricular wall of supraglottic region with superior extension. Thus, extracapsular extension is an important marker for prognosis (18). Therefore, supraglottic area is rich with lymphatic vessels but glottic area is poor in that way. Rodrigo et al. showed higher levels of podoplanin expression in glottic carcinomas (p=0.01) (34). On the other hand, in our study the increase of podoplanin expression was found higher in supraglottic carcinomas than in glottic carcinomas, although the increase of podoplanin expression was obtained in early stages in patients with supraglottic carcinomas (%35) rather than in patients with glottic carcinomas (%53.8). The reason of the difference may be due to the high levels of lymph duct’s plexus localization in supraglottic carcinoma versus glottic carcinoma patients. Recent experimental results have demonstrated that podoplanin mediates a pathway leading to collective cell migration and invasion in vitro (41-42). However, thyroid cartilage invasion depends directly on the primary tumor stage. In addition, the extra capsular spread of the nodal metastasis is related with the tumor stage and the survival rate. 

Glutathione (GSH) has many important cellular functions such as amino acid transportation, maintenance of proteins in a reduced state and cellular protection against reactive oxygen species, drugs and heavy metal ions. The glutathione S-transferases (GST) are a family of detoxification and Glutathione S-transferase T1 (GST-T1) is a subgroup of the glutathione S-transferases (GST) that can metabolize endogenous and exogenous toxins and carcinogens. Some studies have shown that the GST-T1-null genotype was a protective factor against bladder cancer (43). This suggests that decreased GST-T1 enzyme activity may reduce the production of as yet unknown carcinogens. Diedrich et al. reported that GST-T1 transcripts are expressed in neoplastic cells of brain tumour types and O'Shaughnessy et al. reported that GST-T1 was detected at a lower level in 85% of fetal liver sample (44-45). Therefore, GST-T1 gene expression was investigated in the study. However in the present work GST-T1 expression was not observed. many biomarkers were found to determine the prognosis or metastatic disease of many malignancies, but no biological marker was found yet for determination of the survival rate or metastatic disease for laryngeal cancer.
